# Supplementary material for: Stx2 Induces Differential Gene Expression and Disturbs Circadian Rhythm Genes in the Proximal Tubule
Source: Toxins (Basel). 2022 Jan 19;14(2):69. doi: 10.3390/toxins14020069 (PMC8874938; doi:10.3390/toxins14020069)
Supplement: Supplementary file 1 [file toxins-14-00069-s001.zip › Supplemental Table legends.pdf]

**Table S1.** gene annotation and GO_metascape_result. GO terms are listed with corresponding genes.

**Table S2.** Metascape enrichment. Gene symbols are listed to show which genes are within which top 20 GO terms.

**Table S3.** PBS72 normalized log2. PBS (vehicle)-injected 72 h microarray data are compared with 0 h time point and shows all 58 genes are within log2-fold (log2(G2/G1)of between -1 and 1.
