# Supplementary material for: Stx2 Induces Differential Gene Expression and Disturbs Circadian Rhythm Genes in the Proximal Tubule
Source: Toxins (Basel). 2022 Jan 19;14(2):69. doi: 10.3390/toxins14020069 (PMC8874938; doi:10.3390/toxins14020069)
Supplement: Supplementary file 1 [file toxins-14-00069-s001.zip › toxins-1526813 Supplementary Figures.pdf]

# Supplementary Materials: Stx2 Induces Differential Gene Expression and Disturbs Circadian Rhythm Genes in the Proximal Tubule

**Fumiko Obata, Ryo Ozuru, Takahiro Tsuji, Takashi Matsuba and Jun Fujii**

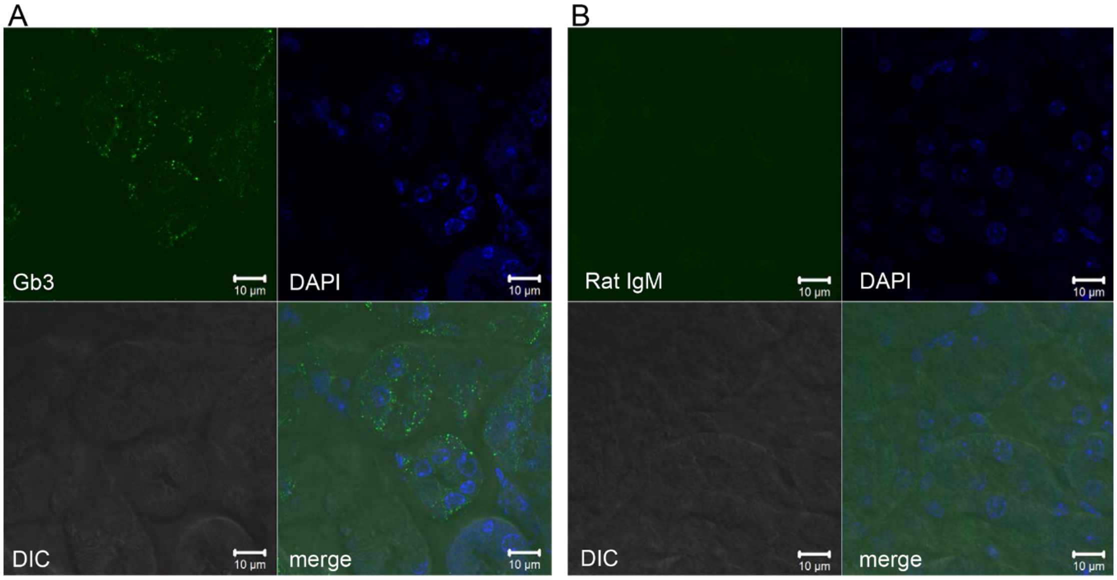


**Figure S1.** The appearance of isotype control (rat IgM) to Gb3 immunofluorescence staining. (**A**) Appearance of mouse kidney free-floating section anti-Gb3 immunofluorescence stain with DAPI, DIC and merged pictures. (**B**) Isotype control (rat IgM) is used in the place of anti-Gb3 antibody to show the negative reaction of normal rat IgM. Rat IgM, DAPI, DIC and merged images.

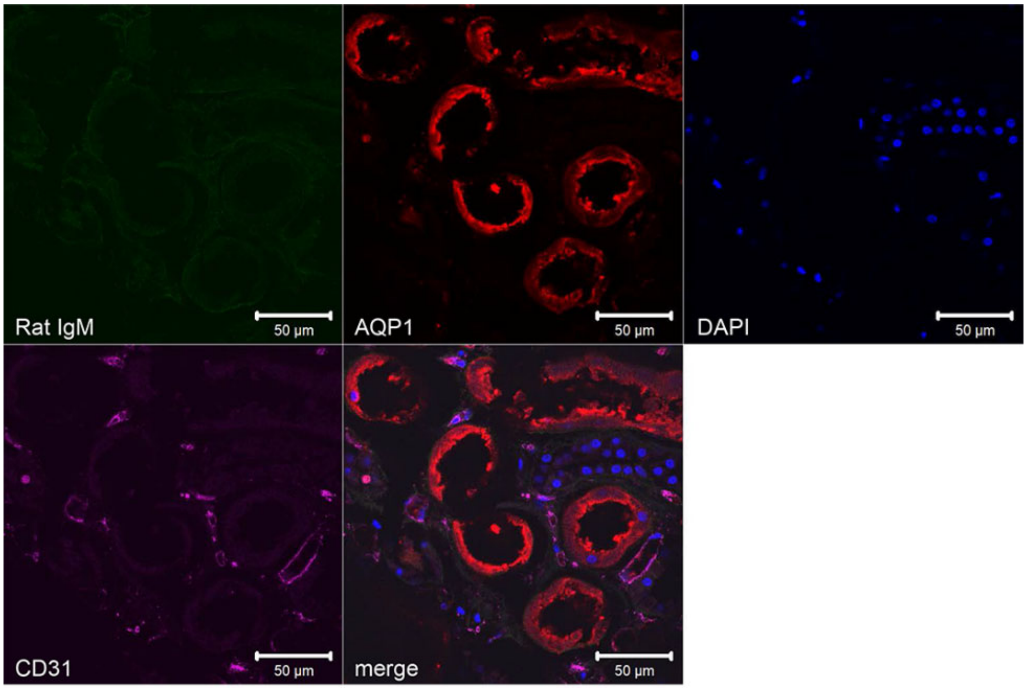


**Figure S2.** The appearance of isotype control (rat IgM) to Gb3 immunofluorescence staining in human kidney. The appearance of human kidney free-floating section isotype control (rat IgM) is used in the place of anti-Gb3 antibody to show the negative reaction of normal rat IgM. Rat IgM, anti-AQP1, DAPI, anti-CD31 immunofluorescence staining, and merged images.

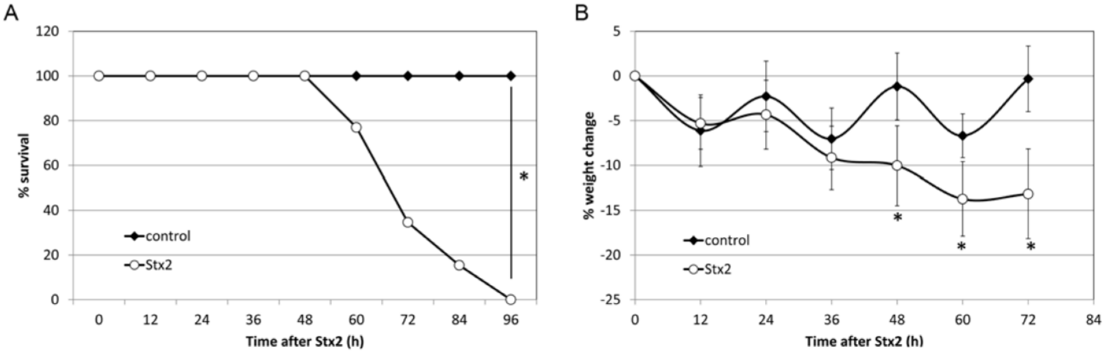


**Figure S3.** Survival curve and weight changes in saline or Stx2-injected mice. (**A**) Percent survival of mice injected with saline (closed circle, control) or with Stx2 (open circle, Stx2). The asterisk denotes statistical significance, $p < 0.05$ in log-rank (Mantel-Cox) test. (**B**) Percent of weight changes in control or Stx2 mice. Asterisks show $p < 0.05$ between saline control and Stx2 in two-way ANOVA followed by Bonferroni test.

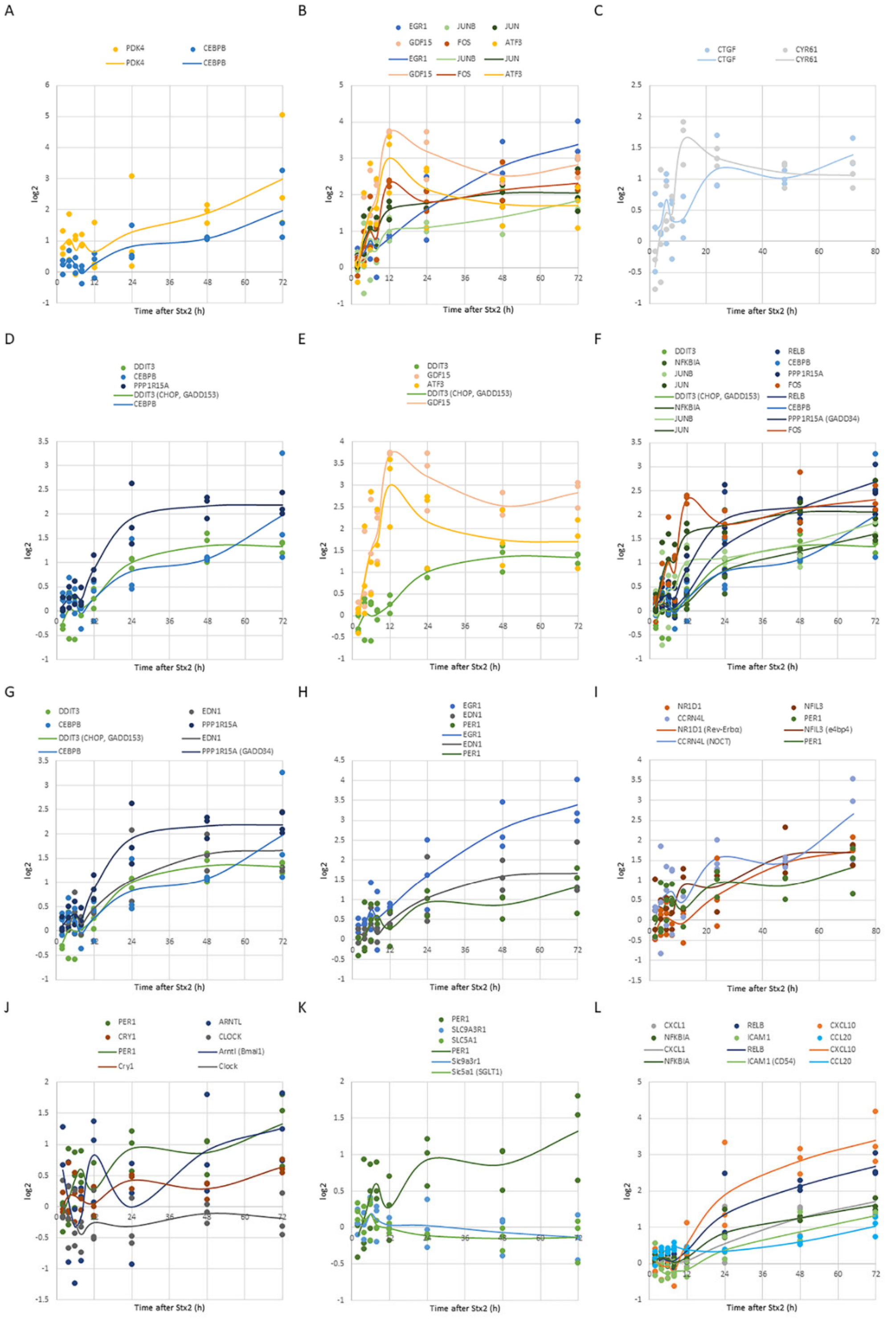


**Figure S4.** Individual datapoints of differentially expressed genes with mouse sampling timeline. Average data trace in Figure 5 is based on these datapoints. Three biological replicates per time point. (**A**) PDK4 and CEBPB mRNA expression patterns are shown. (**B**) immediate-early genes. (**C**) Matricellular proteins CYR61 (CCN1) and CTGF (CCN2) expressions. (**D**) UPR-related genes DDIT3 (CHOP, GADD153), DDIT3 binding partner CEBPB and negative feedback factor PPP1R15A (GADD34) are shown. (**E**) Transcription factors DDIT3 (CHOP, GADD153) and ATF3 and GDF15, which is transcriptionally regulated by these factors, are shown. (**F**) UPR- and RSR-associated genes are shown. (**G**) EDN1 in relation to UPR genes are plotted. (**H**) Early peaks of EDN1 and EGR1 and

following peak of PER1 are shown with later gradual increase in all three genes. (**I**) Genes from figure 4D purple cluster are circadian rhythm-related genes. (**J**) Circadian core genes PER1, Arntl (BMAL1), Cry1 and Clock are shown. (**K**) PER1 and circadian clock-regulated renal genes are shown. (**L**) Inflammatory genes with NF-kB pathway-related genes are shown.

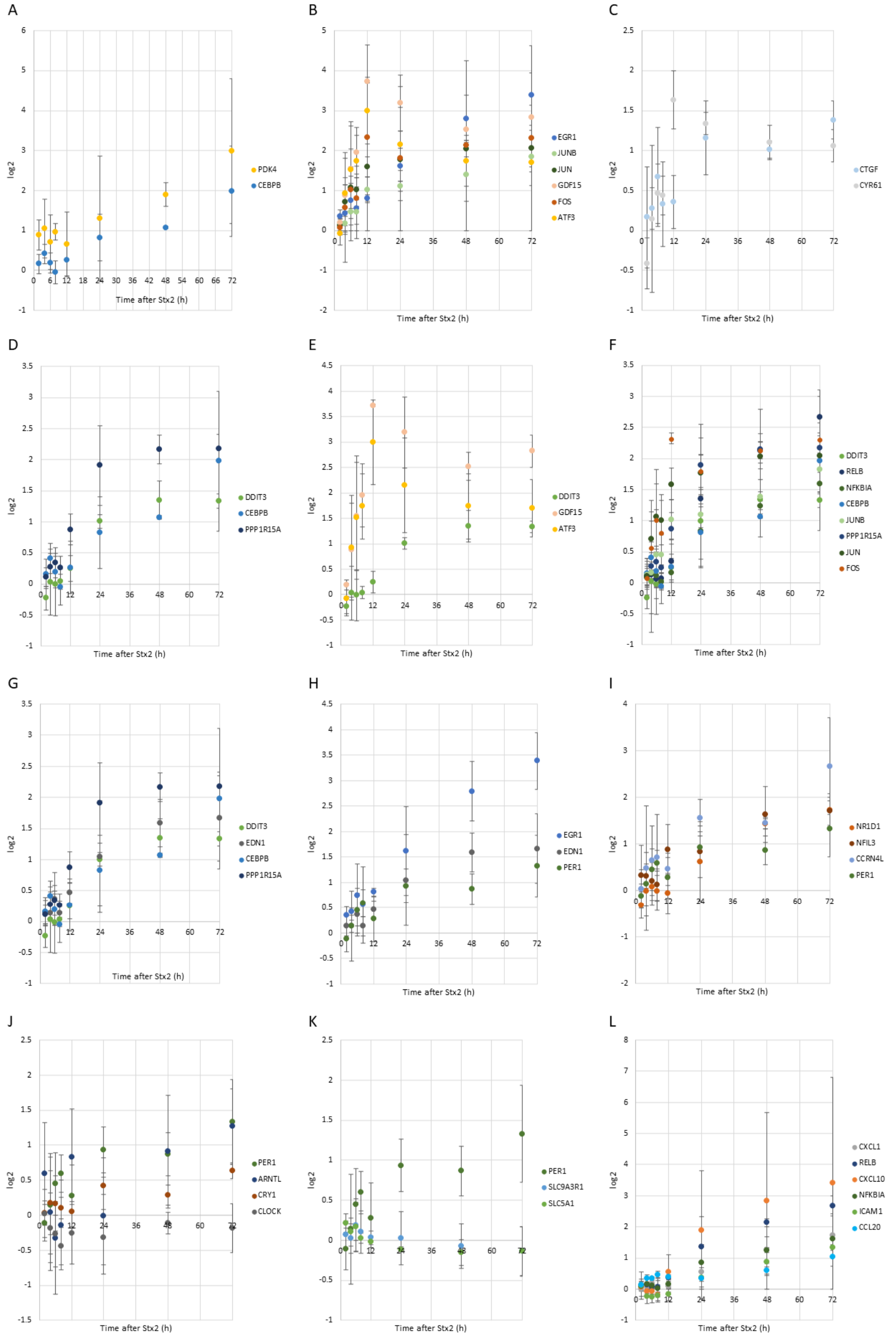


**Figure S5.** Averages and standard deviations of differentially expressed genes with mouse sampling timeline. Average data with standard deviation bars are shown as the same alignment as figure 5 and supplemental figure S4. Three biological replicates per time point. (**A**) PDK4 and CEBPB mRNA

expression patterns are shown. (**B**) immediate-early genes. (**C**) Matricellular proteins CYR61 (CCN1) and CTGF (CCN2) expressions. (**D**) UPR-related genes DDIT3 (CHOP, GADD153), DDIT3 binding partner CEBPB and negative feedback factor PPP1R15A (GADD34) are shown. (**E**) Transcription factors DDIT3 (CHOP, GADD153) and ATF3 and GDF15, which is transcriptionally regulated by these factors, are shown. (**F**) UPR- and RSR-associated genes are shown. (**G**) EDN1 in relation to UPR genes are plotted. (**H**) Early peaks of EDN1 and EGR1 and following peak of PER1 are shown with later gradual increase in all three genes. (**I**) Genes from figure 4D purple cluster are circadian rhythm-related genes. (**J**) Circadian core genes PER1, Arntl (BMAL1), Cry1 and Clock are shown. (**K**) PER1 and circadian clock-regulated renal genes are shown. (**L**) Inflammatory genes with NF-kB pathway-related genes are shown.
